# Instructor feedback versus no instructor feedback on performance in a laparoscopic virtual reality simulator: a randomized educational trial

**DOI:** 10.1186/1472-6920-12-7

**Published:** 2012-02-28

**Authors:** Jeanett Oestergaard, Flemming Bjerrum, Mathilde Maagaard, Per Winkel, Christian Rifbjerg Larsen, Charlotte Ringsted, Christian Gluud, Teodor Grantcharov, Bent Ottesen, Jette Led Soerensen

**Affiliations:** 1 Department of Obstetrics and Gynecology, Juliane Marie Centre, Centre for Women, Children and Reproduction, Rigshospitalet, University Hospital of Copenhagen, Blegdamsvej 9, Copenhagen 2100, Denmark; 2 Department of Obstetrics and Gynecology, Hillerød Hospital, Dyrehavevej 29, Hillerød 3400, Denmark; 3 Copenhagen Trial Unit, University of Copenhagen, Dept, 33.44, Blegdamsvej 3b, Copenhagen 2200, Denmark; 4 Centre of Clinical Education, Rigshospitalet, University Hospital of Copenhagen, Blegdamsvej 9, Copenhagen 2100, Denmark; 5 Department of Surgery, St. Michael's Hospital, University Hospital of Toronto, 30 Bond St., ON M5B 1W8 Toronto, Ontario, Canada

**Keywords:** Virtual reality simulation, Laparoscopy, Training, Salpingectomy, Feedback

## Abstract

**Abstract:**

**Background**

Several studies have found a positive effect on the learning curve as well as the improvement of basic psychomotor skills in the operating room after virtual reality training. Despite this, the majority of surgical and gynecological departments encounter hurdles when implementing this form of training. This is mainly due to lack of knowledge concerning the time and human resources needed to train novice surgeons to an adequate level. The purpose of this trial is to investigate the impact of instructor feedback regarding time, repetitions and self-perception when training complex operational tasks on a virtual reality simulator.

**Methods/Design**

The study population consists of medical students on their 4^th ^to 6^th ^year without prior laparoscopic experience. The study is conducted in a skills laboratory at a centralized university hospital. Based on a sample size estimation 98 participants will be randomized to an intervention group or a control group. Both groups have to achieve a predefined proficiency level when conducting a laparoscopic salpingectomy using a surgical virtual reality simulator. The intervention group receives standardized instructor feedback of 10 to 12 min a maximum of three times. The control group receives no instructor feedback. Both groups receive the automated feedback generated by the virtual reality simulator. The study follows the CONSORT Statement for randomized trials. Main outcome measures are time and repetitions to reach the predefined proficiency level on the simulator. We include focus on potential sex differences, computer gaming experience and self-perception.

**Discussion**

The findings will contribute to a better understanding of optimal training methods in surgical education.

**Trial Registration:**

NCT01497782

## Background

### Virtual reality simulation

For virtual reality (VR) simulation the benefits are clear; the drawbacks are less clear. Throughout the last decade several studies have found positive effects of VR training on the learning curve as well as the improvement of basic psychomotor skills in the operating room, both in surgery and gynecology [1-4]. VR simulators offer standardized and reproducible laparoscopic tasks, ranging from simple basic skills training to complex procedures such as a cholecystectomy or salpingectomy. Despite the well-established advantages of VR simulators, the majority of surgical and gynecological departments encounter hurdles implementing this form of training in surgical education [5]. This is mainly due to lack of knowledge concerning the time, human resources, and costs needed to train novice surgeons to an adequate level [5-7].

Current literature suggests a predefined proficiency level based on experts' performance as a preferred outcome for novice training rather than a fixed training time [8-10], but it has not been investigated whether instructor feedback impacts training to this predefined proficiency level in complex operational tasks. One study suggested that proctored instruction did not offer any advantages to trainees compared with an independent approach [11]; however, the authors investigated only training of simple basic skills, such as instrument coordination, and did not include complex tasks, which is necessary in an advanced surgical training program.

### Self-directed learning

Researchers in motor skills have demonstrated that participants who self-direct their access to instruction or feedback during practice learn more than those whose access is externally controlled [12,13]. It is uncertain whether these results apply in a surgical training environment. One study found self-directed learning beneficial in terms of unlimited access to video instruction, the technical task being simple suturing of a wound [14]. The present randomized trial focuses on self-directed learning in regards to when to receive instructor feedback.

### Research goals

With a worldwide proliferation of simulation centers, it is essential to explore the optimal setting for laparoscopic training and investigate different learning approaches, e.g., a self-directed approach. The overall research goal of the present trial is to compare a feedback guided approach with an independent approach when training operational procedures on a laparoscopic VR simulator. Secondary research goals are to examine self-directed learning towards receiving instructor feedback along with potential sex differences and computer game experience during VR training. With this randomized trial we aim to study the need for human resources and optimal training methods when using VR simulation, and, thus, explore the best set up for surgical education.

We hypothesize that instructor feedback is pivotal when training on a VR simulator and will significantly improve surgical skills and self-perception. Additionally, retention of acquired surgical VR skills will be examined after six months.

## Methods/Design

### Participants

Participants are medical students recruited through advertisements on websites at three student associations (Young Surgeons, Young Anesthesiologist, and the General Student newspaper) at the Copenhagen University Medical School, Copenhagen, Denmark. All volunteers are then invited to an obligatory introduction meeting in order to qualify for the trial.Inclusion *criteria are*: 1) Medical bachelor degree (completion of the first 3 years out of six at University of Copenhagen Medical School). 2) Informed consent before enrollment. *Exclusion criteria are*: 1) having conducted more than 3 independent laparoscopic procedures. 2) Prior experience with VR simulation. 3) Not fluent in the Danish language.

### The VR simulator task and equipment

The VR simulator used is a LapSim^®^, version 2010, produced by Surgical Science, Sweden. Monitor: Dell 22", Simball™ 4D Joystick by G-coder Systems AB with a double footswitch.

All participants will be instructed in the operational technique and use of the VR simulator. The procedure specific VR task is a right-side laparoscopic salpingectomy due to an ectopic pregnancy. At the end of each completed task, the VR simulator sums up a weighted total score based on the values of 11 variables, which reflect time spent and quality of performance and presents an automated feedback on each variable, Table 1. This is available for all participants.

**Table 1:** The 11 variables that generate the automated feedback on the VR simulator

Variable	Passing range
Total Time	> 280 (s)

Blood Loss	> 180 (ml)

Pool Volume	> 10 (ml)

Ovary Diathermy Damage	> 3 (s)

Tube Cut: Uterus Distance	> 4 (mm)

Bleeding Vessel Cut	0

Evacuation from Body	> 1

Left Instrument Path Length	> 2 (m)

Left Instrument Angular Path	> 350 (degrees)

Right Instrument Path Length	> 3 (m)

Right Instrument Angular Path	> 450 (degrees)

The training sessions are repeated until the predefined proficiency level, referred to as the 'expert level', has been reached twice within five consecutive repetitions. The instructor observes correct use of instruments and operation technique. The predefined proficiency level was set and validated in a previous study by the same research group [15], and it has shown to improve surgical skills, such as time and precision, in the operation room [4].

### The experimental intervention group and the control group

*The experimental intervention group *has the option of receiving three feedback sessions from an instructor. The instructor feedback consists of 10 to 12 min of standardized assessment of an operation on the VR simulator and assessment of the automated feedback produced by the VR simulator. The first feedback is given following the first operation on the VR simulator. The participants decide when they want the optional second and third feedback session. One instructor (the main author) provides the feedback in order to ensure consistency and uses the same template for every participant.

*The control group *only receives the automated feedback produced by the VR simulator.

Both groups are asked to complete a questionnaire before and after the trial pertaining to perception of own surgical skills, satisfaction with VR training, and carrier choice.

### Outcome measures

Primary outcome measures are number of repetitions to reach 'expert level' and total time (min) used to reach 'expert level'.

The secondary outcome measure is the weighted total score obtained when 'expert level' is reached which is based on the above mentioned 11 variables, Table 1.

Post hoc analyses will focus on results from the questionnaire reflecting the student's self-perception and the effect of sex and computer gaming skills.

### Ethics

The trial fully complies with the Helsinki Declaration on biomedical research. The Danish National Committee on Biomedical Research Ethics evaluated and approved the trial (journal number: H-3-2010-082). The Danish Data Protection Agency approved collection, analysis and storage of data, approval code 2007-58-0015/30-0996.

All participants are provided with written information on the trial. Participation is voluntary; no material goods are given to participants. The trial is registered at clinicaltrials.gov with trial registration number: NCT01497782.

### Randomization and participation

**Figure 1:**
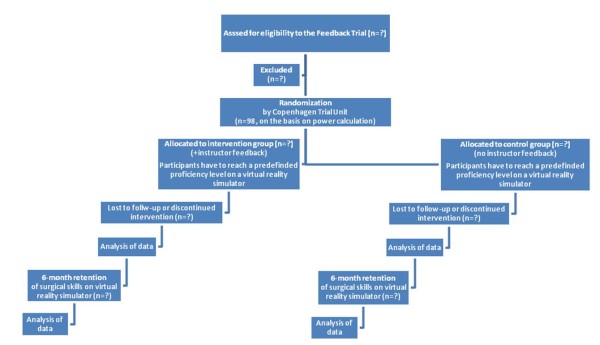
**Enrollment of participants according to the COSORT Statement**.

The central computerized randomization is performed by the Copenhagen Trial Unit. The randomization procedure is concealed and executed by using the participants' unique Central Personal Register number. The trial follows the CONSORT Statement for randomized trials, Figure 1. Stratification variables are sex and computer gaming experience (less than or equal to or more than 20 h annually).

Once the participants are randomized, they can book a VR training time in an online schedule created for the purpose. All participants are given instructions on how to use the VR simulator, e.g., how to enter and exit the task, instrument use, and diathermy use. This is ensured by one of the main investigators (JO and FB). Only these two persons will be in contact with the participants.

Each training session lasts approximately 3 h, and only one training session per day is allowed. The participants have to complete the trial within an 8-week period.

Every time the participants work with the VR simulator, the details and scores for each repetition are electronically recorded. The data will be entered in a secure database by an independent observer. One of the two main investigators is present at all training sessions in the skills laboratory at Rigshospitalet, University Hospital of Copenhagen where the simulator is located.

### Sample size calculation and statistical analysis

Based on data in a previous study [15], we assume that participants in the intervention group and in the control group on the average will use 25 and 40 repetitions, respectively, to reach 'expert level'. With alpha set at 0.05 and beta set at 0.80, the sample size adds up to 98 participants in the trial.

The data will be analyzed using SPSS (Chicago, IL) version 15.0 and the SAS version 9.1. Two sided significance tests will be used.

*Analysis of the first experiment, i.e. instructor feedback approach vs. an independent approach: *The distributions of each outcome measure will be compared between the intervention group and the control group using the general linear uni-variate model and the analyses repeated with the co-variate semester number and the two protocol specified co-factors (indicator of sex, and indicator of computer game experience) included. A post hoc analysis including sex, the intervention indicator(I) and the interaction between I and sex will be conducted to explore the role of the sex.

If the assumptions of the general linear model (normally distributed residuals and variance homogenecity of the groups compared) cannot be fulfilled using simple transformations of the data, the distributions of the intervention group and the control group will be compared using a non-parametric test (Mann-Whitney) as will the two distributions of the results of the questionnaire reflecting the students self-perception at the end of the trial.

To adjust the P values for multiplicity Holm's procedure will be used [16].

#### Missing values

All of the above analyses will be complete case analyses. If a significant effect of the intervention after adjustment for multiplicity is noted three sensitivity analyses will be carried out where increasing degree of bias will be artificially induced by replacing missing values by constructed ones reflecting the degree of scepticism of the observed effect as follows.

#### Worst case scenario

Missing values will be replaced by the most pessimistic value in the opposite group. For time spent e.g. missing values in the group where the best effect was found will be imputed by the maximum value found in the other group and missing values in that group will be imputed by the minimum value found in the first group.

#### Strong bias

Missing values will be imputed by most pessimistic value in the group to which it belongs.

#### Mild bias

Missing values will be imputed by mean value found during the complete case analysis of the group to which the missing value did not belong.

#### Analysis of the second experiment, i.e. the retention of skills

To analyze the data collected during the second experiment comprising time spent and number of repetitions used to reach the expert level by those persons who completed the first experiment the mixed model with repeated measures will be used. An unstructured co-variance matrix will be used. If convergence fails, a choice will be made between the compound symmetric (cs) and the cs plus a random intercept using Akaike's criterion. The basic model will be as follows:

Y=a+bI+ct+dtI

Where I is the indicator of intervention, t is time (time1 and time2 corresponding to experiments 1 and 2 respectively) and a through d are coefficients of the regression equation.

A significant main effect of time indicates that in general retention is present and a significant interaction between I and t indicates that the magnitude of the retention depends on the intervention. The analysis will be repeated with the semester # and the protocol specified stratification variables included as co-variates.

If a significant result is obtained it will be tested in a post hoc analysis if the retention depended on the students' sex or computer game experience (one factor at a time will be included in the model and the possible interactions involving the intervention and time analyzed).

*Missing values *Using the mixed model with repeated measures secures that all observed values are used and that no bias will be present as long as values are missing at random.

To examine the potential effect of bias resulting from values missing not at random a worst case but still not completely implausible scenario is defined as one where each value missing in the second experiment is identical with the corresponding value obtained during the first experiment. The missing values will be imputed accordingly and the analysis then repeated.

## Discussion

### Influence of instructor feedback

The present randomized trial will compare a feedback approach with an independent approach during training of a complex surgical task on a laparoscopic VR simulator. Identifying the impact of instructor feedback may clarify the need for human resources when surgical training encompasses VR simulation. Furthermore, the trial can impart focus on feedback during VR training.

One randomized trial with 36 participants demonstrated that an independent approach to VR training, in which trainees rely solely on automated simulator feedback, required fewer training hours than a proctored approach to achieve simulator proficiency [11]. The trial tested basic laparoscopic tasks on a VR simulator: camera navigation, instrument navigation and coordination, clip applying, and grasping. These tasks are relatively easy to accomplish intuitively, and is very different from the present trial where the task is a complex operational procedure involving both knowledge and motor skills. Furthermore, the trial [11] had an overweight of participants who had had prior laparoscopic exposure in the non-proctored group (equaling our control group), which could affect the results.

Another randomized trial, where the intervention group had access to VR training (but no feedback) and the control group did not train on a VR simulator, indicated that simulator training in a non-supervised setting may not be sufficient to increase laparoscopic suturing skills [17]. The absence of supervised training could conceivably lead to bad habits, such as inappropriate tissue handling and instrument use, which could be detrimental in the operating room.

Several studies have focused on reactions from trainees using simulation-based training, and among the advantages found were: improved self-confidence and self-efficacy, and improved feeling of being proficient [18,19]. However, there were also some indications of drawbacks, which include high levels of anxiety and stress [20,21]. Although little is known about which personal and contextual factors facilitate or impair transfer of learning from a simulation based setting to the clinical setting, it is thought to be important to provide feedback early on in order to support optimal performance [22].

### Self-guided feedback

A self-directed learner takes responsibility for his or her knowledge production by becoming behaviorally and metacognitively active, and increased autonomy probably allows the participant to tailor knowledge production to his or her specific needs [12,14]. Within motor skills learning, researchers have demonstrated that participants who self-direct their access to instruction or feedback during practice learn more than those whose access is externally controlled [12,13,23]. On the basis of these studies, we let the participants in the intervention group decide individually when they want the optional second and third feedback session. To our knowledge, no prior studies have focused on the optimal time to provide feedback in surgical VR training, and expect the present study will provide a guiding principle.

#### Sex differences in surgical VR training

Several studies have found that men spend less time and produce fewer errors in surgical simulator training compared with women, however, this difference remains largely unrecognized and, consequently, unaddressed [24-26]. In an environment where laparoscopic training is increasingly used for evaluation of residents, it is important that surgical curricula acknowledge sex differences to ensure fair and personalized training opportunities [26]. Likewise, this trial will also explore potential sex differences in VR training.

#### Study limitations

None of the participants have performed laparoscopic surgery prior to the study, which could be seen as differing from the group of first-year residents to whom a VR curriculum would apply. In a Danish setting, the participants actually resemble first year residents since they often have no prior laparoscopic training either. The participants are recruited from special interest groups, i.e. students joining technological student organizations. In that respect they resemble residents more than the average student.

It would be optimal with residents as participants, but it is unrealistic since there are only 28 gynaecological residents a year in the Capital and Zealand Region of Denmark.

## Conclusion

We hope this trial will improve our understanding of how to optimize the efficiency of surgical VR training and thus lead to better patient outcomes.

## Abbreviations

VR: Virtual reality.

## Competing interests

The authors declare that they have no competing interests.

## Authors' contributions

JO, FB, MM, PW, CRL, CR, CG, TG, BO and JLS participated in conception and design of the trial. JO and FB are main investigators. JO drafted the manuscript. PW performs all statistics. All authors critically revised the manuscript and read and approved the final manuscript.

## Pre-publication history

The pre-publication history for this paper can be accessed here:

http://www.biomedcentral.com/1472-6920/12/7/prepub
